# SARS-CoV-2 as a Zooanthroponotic Infection: Spillbacks, Secondary Spillovers, and Their Importance

**DOI:** 10.3390/microorganisms10112166

**Published:** 2022-10-31

**Authors:** Georgios Pappas, Despoina Vokou, Ioannis Sainis, John M. Halley

**Affiliations:** 1 Institute of Continuing Medical Education of Ioannina, 45333 Ioannina, Greece; 2 Department of Ecology, School of Biology, Aristotle University of Thessaloniki, 54124 Thessaloniki, Greece; 3 Medical School, Faculty of Health Sciences, University of Ioannina, 45110 Ioannina, Greece; 4 Laboratory of Ecology, Department of Biological Applications and Technology, Faculty of Health Sciences, University of Ioannina, 45110 Ioannina, Greece

**Keywords:** zooanthroponotic infection, spillback, spillover, mink, white-tailed deer, zoo outbreaks, SARS-CoV-2, animals

## Abstract

In the midst of a persistent pandemic of a probable zoonotic origin, one needs to constantly evaluate the interplay of SARS-CoV-2 (severe acute respiratory syndrome-related coronavirus-2) with animal populations. Animals can get infected from humans, and certain species, including mink and white-tailed deer, exhibit considerable animal-to-animal transmission resulting in potential endemicity, mutation pressure, and possible secondary spillover to humans. We attempt a comprehensive review of the available data on animal species infected by SARS-CoV-2, as presented in the scientific literature and official reports of relevant organizations. We further evaluate the lessons humans should learn from mink outbreaks, white-tailed deer endemicity, zoo outbreaks, the threat for certain species conservation, the possible implication of rodents in the evolution of novel variants such as Omicron, and the potential role of pets as animal reservoirs of the virus. Finally, we outline the need for a broader approach to the pandemic and epidemics, in general, incorporating the principles of One Health and Planetary Health.

## 1. Introduction

Most emerging and re-emerging infections of recent decades are zoonotic [1]. They comprise the majority of the recent important infectious disease outbreaks worldwide, including the still evolving SARS-CoV-2 (severe acute respiratory syndrome-related coronavirus-2) pandemic. Several alternative explanations, requiring further clarification, have been proposed for the still obscure origin of the virus, but a zoonotic origin remains the leading hypothesis [2,3]. Other coronaviruses with major morbidity and mortality potentials that have emerged in the 21st century are SARS-CoV (the “godfather” of the Sarbecovirus group where SARS-CoV-2 also belongs) [4] and MERS-CoV (Middle East Respiratory Syndrome-related coronavirus, also a beta-coronavirus, but of the Merbecovirus group) [5]; for both viruses, bats have been outlined as the natural reservoir, while palm civets and camels, respectively, were deemed to be intermediate hosts between bats and humans. In a similar fashion, given that SARS-CoV-2 bears considerable genomic homology to other sarbecoviruses found in bats of Chinese provenance and from neighboring countries such as Laos [6], one can presume that SARS-CoV-2 originated in bats and then, directly, or through a still undetermined, intermediate host, succeeded in spilling over to humans, thereafter resulting to a pandemic, sustained through human to human transmission. 

When discussing the eradication of infectious diseases worldwide following the “smallpox-eradication” mode, the absence of animal reservoirs of the specific pathogens is often considered a prerequisite [7]. During the current pandemic, viral eradication was never actually on the table (notwithstanding the unexpected disappearance of SARS-CoV in 2004 [8]) for reasons unrelated to animal reservoirs. Yet, when a pathogen such as the SARS-CoV-2 emerges and manages to infect hundreds of millions of humans, and eventually mutates into novel variants that perpetuate its spread [9] and secures its path to endemicity, a major question is going to be where the virus resides. This is because “where SARS-CoV-2 resides”, be it an immunocompromised human or a susceptible animal population, will be also where the virus mutates into variants that could be more effective, more transmissible, and more capable of immune escape.

**Figure 1:**
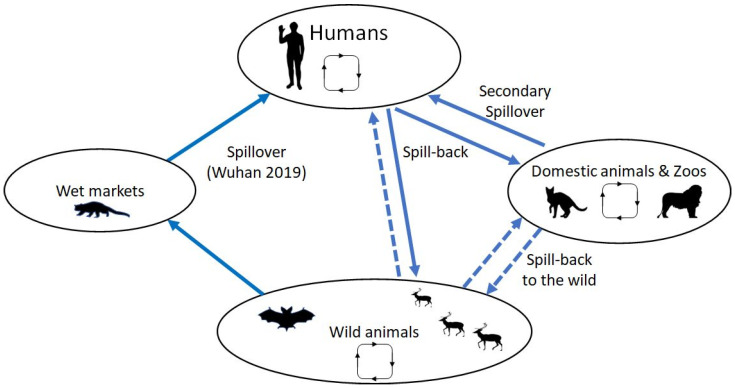
Schematic diagram for the known and inferred transmission pathways for SARS-CoV-2. The virus originated in the wild, probably in bats, and initial spillover/amplification happened at the Wuhan Seafood wet market. Entering the human population, the virus caused the COVID-19 pandemic. The resulting circulation led to the evolution of many new variants. Spillback into domestic and zoo animals was observed beginning in 2020, notably with mink in Denmark, where circulation in the farm populations led to new strains. Direct spillback from humans into wild animals has been observed, also followed by circulation and evolution of new strains. Secondary spillover from the wild into domestic animals or humans and/or spillback into the wild from captive animals are denoted by broken lines (animal silhouettes were obtained from Wikimedia commons).

In the present review, we attempt an evaluation of the extent of zooanthroponotic transmission of SARS-CoV-2, that is transmission from humans to animals, as well as of the gaps in our understanding of this reverse spillover (Figure 1). We also discuss the importance of such episodes and the possible ensuing pathogen endemicity in animal populations and spillover into humans again. In order to record all cases of zooanthroponotic transmission, we searched medical literature databases (PubMed, Scopus, and Google Scholar) using the search term “SARS-CoV-2” and a series of other terms, including individual common animal names (e.g., cats, dogs, bats, etc.) or other terms corresponding to animal groups of higher order than that of species (e.g., Felidae, Canidae, Mustelidae, etc.). All articles were evaluated by two of the authors for the relevance of their content. Articles reporting experimental laboratory infection of animal species were not included, as they were beyond the scope of this article. We further searched the relevant reports of the World Organization of Animal Health [10] to identify transmission events, possibly not reported yet in scientific literature, and we also performed a similar search in SARS-ANI, an open-access dataset of similar events, developed by scientists from the University of Veterinary Medicine of Vienna, Austria, the Complexity Science Hub, and the Wildlife Conservation Society [11]. Cumulative data, thus collected until 2 October 2022, were used in preparing this manuscript. Table 1 depicts all known animal infections of potential zooanthroponotic origin. As counts of animal cases would be a gross underestimate, we decided to broadly depict the known extent of the effect of this transmission and the subsequent intraspecies circulation.

SARS-CoV-2 is often described as a “generalist” pathogen. The ability to infect multiple living species is recognized on theoretical grounds, on the basis of the predominant receptor that the virus attaches to in host species. This is the agiotensin-converting enzyme 2 (ACE2) receptor, which is abundantly observed in the animal kingdom, apart from humans [50]. Early in the pandemic, isolated reports of zooanthroponotic transmission of SARS-CoV-2 emerged, particularly in domestic cats and dogs [51,52]. As the pandemic proceeded, it became obvious that the extent of such events was great and involved many different species of animals.

## 2. What the Mink Taught Us

The first animal population that emerged with a role in sustained intraspecies transmission and secondary spillover potential was the mink. Numerous outbreaks in mink farms of Europe and the US were reported [53,54,55]; the first outbreaks were observed in the Netherlands, where the majority of the 126 mink farms were diagnosed with SARS-CoV-2 infections in the period from April 2020 to November 2020. Initial infection of these animals, presumably by humans working in such farms, was followed by sustained animal to animal transmission facilitated by the dense confinement of the caged animals. This intense intraspecies transmission resulted in the subsequent emergence of novel variants that then spilled over to humans again. In Denmark, the identification of such a mink-originating variant (cluster 5), transmissible to humans, was troubling in terms of immune evasion and led to the decision for a mass cull of mink in order to minimize further risk to humans [56,57]; at that time, a significant percentage of the human-infecting SARS-CoV-2 strains in Denmark were mink-derived, indicating a generalized secondary zoonotic transmission event. The decision to cull all mink in Danish farms and to suspend function of all mink farms was the final step in mitigation efforts that started with localized mink culling and continued with aggressive surveillance and regional culling (i.e., culling at all mink farms located in an area surrounding one with cases, in a radius of 7.8 km). When these policies failed in containing the extended circulation of mink-derived strains in the community, and when cluster 5 appeared, a decision for mass culling was unavoidable. This decision raised ethical issues, similar to those seen in the UK during the BSE crisis in the 1980s, where 4.4 million cattle were culled, as well as highlighting already-existing ethical concerns about such farms [58,59]. Similar outbreaks seem to have continued throughout the pandemic, often almost cryptically; Greece is a typical example, where numerous outbreaks in mink farms in the northwestern part of the country were recorded early on and were still being recorded during the second pandemic year, till the summer of 2021 at least, with a total mink death toll due to SARS-CoV-2 infection exceeding 1000. Yet, information about these outbreaks has only sparsely been presented in the scientific literature [60], and they have largely been ignored by mass media. One has to consult the relevant reports of the World Organization for Animal Health [61] to appreciate the magnitude of these outbreaks. However, in this country, a more constrained approach towards outbreak mitigation was adopted, with no mass culling in infected farms.

The Greek mink SARS-CoV-2 outbreaks reveal an overall understudied parameter of the zoonotic potential of the virus: its further spillover, from farm animals to wildlife. The virus may escape from such farms and become a wildlife mink pathogen; repeated episodes of mink escapes (sometimes assisted by activists) [62] have taken place in the recent past in Northwestern Greece, resulting in a sustainable population of free-ranging mink in the area. Transmission from farm-kept mink to semi-domestic animals (such as stray cats) has already been demonstrated in the Netherlands [63], and feral mink infection has been already reported from Spain [64]. The mink outbreaks and their resolution can further raise ecological and other issues. For instance, in response to the furor caused both by the emergence of variants of concern for humans and the eventual animal culling, some European countries announced that all mink farms would cease to exist; in fact, farms were relocated in other European countries that might presumably have more lenient veterinary and public health regulations [65].

Russian scientists developed an inactivated SARS-CoV-2 animal vaccine (with aluminum adjuvant) called Carnivac-Cov/Karnivak-Kov [66], that would allow for an uninhibited production line of the fur industry (where mink farms are utilized) and the safety of pet animals such as cats and dogs. In clinical trials performed at fur farms, animal shelters, and veterinary clinics, the vaccine has proved effective in sustained (for at least six months) seropositivity (as evaluated in mink, dogs, cats, Arctic foxes, and foxes), with further monitoring not raising safety concerns. The feasibility of mass vaccination of such susceptible animals and the cost-effectiveness of such an approach remain unknown, as is its implementation and current field efficacy. Another concern of unknown magnitude is its ability to drive the evolution of resistant variants. At least two other vaccines have been in/post development: the Zoetis company recombinant vaccine for wild animals in zoos and sanctuaries, which had been used in the Illinois zoo, where numerous animal species subsequently tested positive [16], and the vaccine developed from Applied DNA Sciences and EviVax, for companion animals and potentially for other animal species, encouraging initial immunogenicity studies were performed in cats [67]. 

## 3. What the Zoos Taught Us

The next major story of animal infection during the pandemic concerned animals in captivity, including the much-reported cases of the New York Zoo lions and tigers [35]. During the pandemic, many more clusters of cases were reported from all over the world with several animal species involved [16,28,33,36,37,38,39,47,48]. Animals implicated in zoo transmission events include the gorilla, puma, hippopotamus, snow leopard, hyena, otter, etc., (Table 1). Some of the major zoo SARS-CoV-2 outbreaks involving several individuals and, in most cases, more than one species/subspecies are as follows. 

The first zoo outbreak reported was at the Wildlife Conservation Society’s Bronx Zoo in New York City, New York, when a Malayan tiger (*Panthera tigris jacksoni*) with a persistent cough turned out positive in SARS-CoV-2 real-time reverse transcriptase PCR (rRT-PCR) tests. In the following week, six more animals tested positive: another Malayan tiger, two Amur tigers (*Panthera tigris altaica*), and three African lions (*Panthera leo krugeri*). The tigers were housed in the same zoo structure but in different enclosures to the initial case; an additional tiger located in the same zoo structure was not infected. The lions were located in a different zoo structure. During this period, the zoo had already shut down due to the lockdown measures. Thus, animal handlers were considered as the obvious route of infection, with four out of twelve personnel individuals exhibiting active or recent SARS-CoV-2 positivity; two tiger-contacting personnel were rt-PCR positive, indicating active infection, and two lion-contacting personnel were antibody positive, indicating a recent, but not active infection. All four individuals reported symptoms in the preceding days, but also reported that they did not contact the animals, and were confined at home, during their symptomatic days. Further genotype sequencing was performed in samples from infected humans and animals and the results demonstrated that tigers and lions were infected from strains belonging to different clades. Thus, there were two independent spillback events. The tiger-isolated strain was similar to the human strain, demonstrating the initial route of transmission, from human to animal. The other tigers, given the latent period to the development of their infection, seem to have been infected from the initial tiger, since they were housed in the same zoo structure. This initial zoo outbreak led to awareness of the potential for SARS-CoV-2 spillback to caged wildlife, and thus to the development of regulations regarding the use of personal protective equipment by zoo animal handlers. 

Despite these regulations, further zoo outbreaks emerged. In July 2020, a puma was diagnosed positive in a private zoo in Johannesburg, Gauteng, South Africa. Eleven months later, in the same zoo, another puma developed symptoms and was diagnosed, along with three lions; one lion developed symptoms four days after the other two, possibly infected through a different route, since they were housed in non-adjacent zoo structures. Despite the absence of symptoms in all human personnel of the zoo during and shortly before the animal diagnosis, one individual handler tested positive, and genomic sequencing demonstrated an identical isolate both in human and infected animal samples [36]. 

The Tennessee zoo outbreak of October 2020 [47] involved three Malayan tigers. A subsequent epidemiologic surveillance of all human personnel in contact with these animals, in a time period of two weeks before and two weeks after the initial tiger case detection, showed that two out of eighteen individuals were SARS-CoV-2 positive; when restructuring a chronological chain of events, the possibility of a secondary spillover was raised, since a veterinary assistant tested positive after assisting in care of already infected animals. Nevertheless, a tiger keeper that also tested positive after the initial animal case detection could have been infected by a member of their family since a positive household case was already noted. 

The Barcelona zoo cluster in November 2020 involved four Southwest African lions (*Panthera leo bleyenberghi)* that developed symptoms shortly after a cluster of cases was initiated among their human handlers, with the chronological chain of events suggesting an initial spillback event from a pre-symptomatic human to a lion, as genomic sequences of humans and animals were identical [37]. 

A multi-species outbreak took place in February 2021 in the Prague Zoological Garden [28]. Positive animals included the following: six out of eight western lowland gorillas (*Gorilla gorilla gorilla*), at least one of the Asiatic lions (*Panthera leo persica*) out of three (they were pool-sampled, thus, it was not clear whether one, two, or all of them were positive), one out of two Amur leopard cats (*Panthera bengalensis euptilurus*), both Malayan tigers, and one out of two Sumatran tigers (*Panthera tigris sumatrae)*. Gorilla and felid infections likely resulted from different human to animal introductions, with one gorilla handler and two cat handlers testing positive in the immediately preceding period of the initial animal case detection (the zoo was closed for the public during these months). The Alpha variant was implicated in all cases. 

In India, during the Delta variant outbreak in the Spring of 2021, independent cases in lions were noted [38,39]. The largest incident was reported in “Arignar Anna Zoological Park” in Chennai, with nine out of thirteen Asiatic lions turning out positive. Seven of these animals shared a common living environment (also with another two individuals that remained negative), indicating possible animal to animal transmission after an unknown human to animal introduction; the other two positive lions were housed in a different common zoo structure. Two of the infected lions eventually died. The second cluster was in the Lion Safari Park, in Etawah, Uttar Pradesh, in two female lions, located in neighboring premises. The remaining lions of the facility remained asymptomatic and negative. The third incident was in the Nahargarh Biological Park, Jaipur, Rajasthan, with a single positive case in an animal, while all other lions of the facility remained negative. All human personnel tested negative and did not report any suspicious symptoms; yet, in the Etawah cluster, a veterinarian developed symptoms and tested positive two days after tending one of the infected animals (despite the presumed use of personal protective equipment). This is one of the still limited but worrisome cases of secondary spillover, similar to the ones described in the mink-related chapter. 

Another multispecies animal outbreak was in the Chicago Zoological Society’s Brookfield Zoo, Illinois, US, in September 2021, shortly after the initiation of wide animal vaccination with the experimental Zoetis vaccine [16]. The outbreak lasted for two months and involved numerous animal species, including two binturong (*Arctictis binturong*), a fishing cat (*Prionailurus viverrinus*), two lions (unspecified), three snow leopards (*Panthera uncia*), two white-nosed coati (*Nasua nasua)*, and two tigers (an Amur tiger and an unspecified one). Routes of animal infection were not clarified, although there was a possible secondary zoonotic transmission, with an animal handler testing positive shortly after taking care of infected animals. Phylogenetic analysis demonstrated homology between the different animal isolates, indicating a single, unknown, human to animal introduction and a possible subsequent animal to animal transmission. 

Zoo transmission events are usually poised to be isolated or emerging in small clusters, since the compartmentalization of captive animal grounds precludes evolution of such clusters to ecologically significant events. Whether these clusters would translate into a sustained viral circulation in a wildlife population of the same or similar animal species remains unknown; the major bottleneck in such a spillback would be the initial transmission event, in cases where animal species that are not adequately friendly to humans and would need to get infected through environmental contamination (carcasses, wastewater). Animals in zoos are typically infected by the zoo personnel, handlers, or feeders (as in the case of the New York Zoo tigers). However, for more human-friendly animals, infection from unidentified visitors cannot be excluded, resulting in a higher chance of the virus being transmitted to animals. 

## 4. What the Deer Taught Us

White-tailed deer (*Odocoileus virginianus*) are the animals where SARS-CoV-2 managed to accommodate itself most efficiently. Genomic surveillance can attest to the fact that deer were multiply infected from humans [68]. Since the initial reports [69], positivity rates have been systematically high (increasingly in 2021 compared to 2020) in white deer populations of several areas of US and Canada [70,71,72,73], signifying intraspecies transmission, which has been further characterized; male deer seem to be more susceptible, possibly due to their socializing habits during breeding [74] (females and fawns tend to get secluded while males join other males in grazing). The most worrying aspects of viral circulation in the white deer population are the emergence of peculiar SARS-CoV-2 variants from this population [70,75], the persistence of past variants of concern even long after they have ceased to be detected in humans [74], and further spillover to humans [75]. The latter event underlines the general zoonotic risks associated with hunting. Discovering the viral circulation in deer and being able to characterize the virus was a fortunate accident, because white-tailed deer have been consistently sampled for other reasons. These animals could theoretically serve as another intermediate host of importance: their proximity to human life may mean that they could continue to be susceptible to every novel variant of concern circulating in humans; reverse spillovers may then lead to the emergence of novel variants in humans, but also to further wildlife interspecies transmission, given the white-tailed deer habitat is at the junction between domestic life and wildlife. In this vein, there has been speculation about possible transmission of the pathogen from deer to deer ticks (*Ixodes scapularis*), and then back to humans. However, this pathophysiological scenario seems extraordinarily unlikely, since it would demand ticks getting infected through a blood meal by deer (but is infected deer viremia a common event?), and then for the virus to be able to replicate in ticks and end in their salivary glands [76]. 

Viral endemicity in North American white-tailed deer signifies one of the most notable shortcomings in SARS-CoV-2 surveillance; if such an abundant circulation of a pathogen that was systematically investigated was only accidentally discovered, how can we be certain that something similar is not already happening in other animal species which are not sampled? White-tailed deer, for example, can be found in Central and South America, too, but it is not studied there. Whether SARS-CoV-2 has also spilled back in these areas should have already been investigated. Other deer species should also be evaluated, as for example the mule deer (*O. hemionus*), where isolated positives have been reported [22]. The same holds for other deer species whose ACE2 receptors bear a high resemblance to human ACE2 and might thus lead to spillbacks, at least on theoretical grounds [50]. Reindeer or caribou (*Rangifer tarandus*) are predominantly found in North America, Siberia, and northern Arctic regions of Scandinavia, while Pere David’s deer or milu (*Elaphurus davidianus*) are native species of Chinese regions. These populations and species warrant further investigation. 

Other wildlife animal hosts of the virus are the big hairy armadillos (*Chaetophractus villosus*) of the Argentinean pampas. They were reported [13] both as animals that can move freely between human and wildlife habitats, and as reservoirs of variants of concern that have long ceased to circulate between humans (the Gamma variant in the armadillos-case). The grave observation of Arteaga et al. [13] regarding the armadillo presence in cemeteries and the possibility of these omnivorous animals feeding on human corpses provides a potential transmission mode of the virus between humans and these animals. Because certain rodent species also cross wild and domestic habitats, they are suspects of behaving as transmission agents, hence, also requiring further attention. 

## 5. What the Rodents Taught Us

The appearance of the Omicron variant in November 2021, characterized in samples from South Africa and Botswana, took the scientific world by surprise, since this was a variant drastically distant from both the virus wild-type and from the variants of concern known at the time. One of the theories for its origin [77], based on the projected effect of some of its point mutations, was that Omicron BA.1 emerged from mice, which were initially infected from humans (or from human-related sources of the virus such as from wastewater). The theory posits an intense pathogen re-circulation, continued among mice for a prolonged period of time (under the human radar), leading to adaptive mutations and then to a novel variant that subsequently spilled over to humans again. Other experts [78] argue that this was not eventually the case and that initial Omicron variants emerged from prolonged positivity of immunocompromised humans. Given these unresolved issues of high importance, enhanced surveillance is urgently needed. 

This need for surveillance becomes even more urgent, if another human–rodent–virus episode is taken into consideration. A transcontinental Omicron variant transmission from humans to rodents (pets) was reported, originating from the Netherlands, with the infected animals being subsequently transferred (along with the virus) and sold in Hong Kong, where animal to human transmission took place [79,80] resulting in more than 80 human cases, all epidemiologically linked to the imported hamsters, and all attributed to the Delta variant, at a time when this specific variant had disappeared from Hong Kong. It should be noted that the specific Delta lineage had only isolatedly been reported in Hong Kong, with the last case in a traveler that was subsequently quarantined weeks before this particular outbreak. Furthermore, this was a period when the Omicron variant was rapidly expanding in the community. Two batches of Golden Syrian hamsters (*Mesocricetus auratus*) were imported from the Netherlands to Hong Kong in December 2021 and January 2022. After initially being stationed at a specific warehouse, the hamsters were then sold to different pet shops. The first pet shop-related human outbreak involved a pet shop worker and a female customer visiting with her daughter. The female customer was also infected and further infected her husband, with the rest of the household eventually infected (the son and daughter, both asymptomatic; it is not clear whether the daughter was infected in the household or during her visit to the pet shop, accompanying her mother). Seven secondary human cases resulted from exposure to the infected members of this household. Subsequently, transmission chains emerged, originating from three pet shops; in one of them, a pet shop worker secondarily infected their co-workers, a family member, and individuals dwelling in the same housing complex, in a cluster of at least 22 cases. Hamster culling was eventually performed, in order to avoid further transmission chains that would allow the Delta variant to re-enter the Hong Kong community. 

Of note, none of the other animals that were transferred from the Netherlands with the hamsters and were stationed with them in the warehouse turned out positive. These animal species included chinchillas, rabbits, and dwarf hamsters. 

A recent French study reported infection of domestic pet rats by their owner [81].

The pet industry has long been recognized as a means of efficient zoonotic transmission, to which has been attributed [82] the first monkeypox outbreak in the Western World, in 2003. 

## 6. What Our Pets Taught Us

Ever since the initial recognition of SARS-CoV-2 transmission from humans to household dogs and cats [50,51], three important issues arose: whether these domestic animals could sustain intraspecies transmission, whether they could participate in secondary spillover events, and whether further animal transmission, outside the household, could take place. 

Cats and dogs are an integral part of the household where they belong. Several small epidemiological studies recently reviewed regarding cats [18], demonstrated that they get infected after their human owner contracts SARS-CoV-2, with varying positivity rates. Food sharing between humans and pets has been shown as a risk factor for animal infection [83]. 

Given that the cat’s viral receptor ACE2 (angiotensin converting enzyme 2) bears significant resemblance to the human ACE2 receptor [84], high domestic cat seropositivity would be expected. This reached 52% of samples in the Canadian study that reported the highest seropositivity rates [85] (also reporting 41% of samples for the household dog). 

Owner to pet transmission remains the predominant, almost exclusive means of pet infection. A recent Portuguese study [86] demonstrated that pet (cat and dog) positivity correlated to household human positivity. In a household with other pets, animal to animal transmission seems unavoidable [86] and has indeed been documented [86], yet other case reports often demonstrate the opposite [87]. Experience from studies in shelter cats and dogs, living in an environment with intense transmission potential, have shown extremely low infection rates [88,89]. In addition, there is considerable heterogeneity in studies evaluating the duration of infectivity of domestic animals. For example, a Greek study [87] estimated an infectivity period of seven days, a French study considered infectivity as a transient event of weak potential [90], a Chilean study estimated a period ranging from less than a week to more than two weeks [91], while pre-symptomatic positivity had also been reported in the case of an immunocompromised cat [92]. 

Seroprevalence studies evaluating antibody positivity usually evaluate a specific period in a specific area and cannot offer reliable information on differences in seropositivity between countries or even continents, since they are expectedly affected by the general overall pandemic trends of the specific area and time. Thus, such studies are not further discussed here. 

A few studies have evaluated the positivity of stray cats and dogs that could potentially have been infected through interaction with domestic cats and dogs. Such studies, from Italy and Spain, have shown limited viral circulation in cats (given that these stray animal populations have an extended contact network) [93,94]. However, studies for stray dogs in Ecuador (an area with extremely high viral circulation among humans) showed higher positivity [95]. 

On the other hand, domestic cats and dogs have not proven an important vector for secondary spillover to humans. There is only one isolated case report of a human getting infected by a cat (a veterinarian sneezed upon by an infected cat of an infected owner) [96]. Transmission to veterinarians from infected dogs has also been implied in a Nigerian study [97]. This is expected since household pets of infected persons tend to be isolated along with their owners. Thus, they are unlikely to come into contact with other humans, apart from the already-exposed ones in the household (for household contacts though, one cannot easily delineate the exact transmission pathways). 

A subunit vaccine specifically for dogs has been recently developed [98].

## 7. What Animals (Have Not) Taught Us

The 18 March 2022, joint statement by the World Health Organization, World Organization of Animal Health, and the Food and Agricultural Organization [99] stresses the need for continuous animal surveillance studies and public education on the perils and pathways of SARS_CoV-2 spillback. In order, though, to successfully implement such a holistic approach, humans should re-evaluate their overall approach to the ecosystem. 

One of the major drivers of continuing zoonoses emergence is our anthropocentric model of thinking regarding the world around us. Human intrusion into natural habitats allows for enhanced interface between unknown pathogens and us. This intrusion is exemplified, par excellence, through deforestation. An unknown pathogen that resides deep in a forest, in its natural animal reservoir, may never contact humans. However, when this habitat is altered by humans, the natural environment of this animal reservoir is altered, and so is the natural environment of the pathogen. This has been the case for outbreaks of the Nipah and Hendra viruses in Southeast Asia and Australia, respectively, where their natural reservoirs, bats, were forced to feed from fruit trees in direct contact with intermediate animal reservoirs (pigs and horses, respectively), facilitating eventual human infection [100]. In fact, every human intervention that changes the animal habitats may also affect the pathogens they carry. Apart from the environmental changes, an important driver of zoonotic outbreaks has consistently proven to be illegal animal trade; one has to remember that the initial SARS virus presumably entered the human horizon through animals sold in such markets and also that the Huanan Seafood Market in Wuhan has been considered as the generator, or at least a major initial amplifier, of the current SARS-CoV-2 pandemic [2].

Currently, viral diversity is beyond our understanding [101]. We are aware of only a small fraction of the existing pathogens, many of which may theoretically possess zoonotic potential; this is the case, for example, with some of the sarbecoviruses that were recently identified in bats in Laos [6]. Let us remember what our awareness about coronaviruses was only twenty years ago: we then only knew about benign seasonal coronaviruses that partly caused the “common cold”, and we were not aware of any coronavirus that could infect humans with significant morbidity and mortality. Two decades later, coronaviruses have emerged as leading candidates for a next pandemic, let alone the huge toll of the current pandemic.

The recognized zoonotic pathogens of bacterial, viral, parasitic, fungal, or prion nature [102,103] impose major morbidity and mortality as well as a heavy socioeconomic burden. We have barely managed to acquaint ourselves scientifically with the notion of One Health, where a zoonotic infection is viewed through a collaborative approach consisting not only of infectious disease and microbiology specialists, but also of veterinarians, public health specialists, and eventually policy makers [104]. Imagine how demanding the transition to the notion of Planetary Health would be [105]. The concept of Planetary Health views the environment as a continuum, of which humans are only a part, but not its epicenter, and acknowledges that human actions can consistently throw into disarray the ecosystem’s equilibrium. Zoonotic pathogen emergence is only an aspect of this disequilibrium, and its consequences typically extend beyond the human burden of disease [106]. These simple “ecological health” axioms have been largely ignored before and all through the pandemic, and continue to do so. 

Zooanthroponotic transmission of infectious agents, though happening almost certainly extensively, has been notoriously understudied; there have been limited and mostly isolated reports of such transmission [107,108]. This is particularly due to our anthropocentric model of pursuing related research without taking into consideration its ecological context. More specifically, we tend to be interested in studying disease prevalence in humans and often ignore the wider context that includes the evolution of pathogens in different animal hosts, their impacts on species and communities, and the ecological factors that may lead to increased pathogen fitness. A 2014 review of spillback zooanthroponotic events [108] identified cases attributed to bacterial, viral, parasitic and fungal pathogens, and involving wildlife or domestic companion animals. The vast majority of these rare events, though of limited extent, was in contrast to what has been observed with SARS-CoV-2 spillback, where further, sustained, animal to animal transmission has been successful, at least in the case of mink and white-tailed deer. This discrepancy could be attributed to the vastly increased epidemiological research performed during the current pandemic; on the other hand, it may underline the long-term burden of SARS-CoV-2 in Planetary Health, a burden of which we remain unaware. Thus, animal surveillance during the SARS-CoV-2 pandemic may result in a paradox: it may be a case of extremely enhanced study of reverse zoonotic events (compared to other outbreaks/epidemics/pandemics), but at the same time it may also be an extremely understudied aspect of the same pandemic, with further viral circulation in animal populations that happens now unidentified. 

In this framework, one should take into account the potential role of viral adaptation to animal species in emergence of novel variants of human interest and infection dynamic. In the context of mink and deer infection, the “generalist” nature of SARS-CoV-2 allowed adaptation with minimal mutations [109,110,111]. This ease of adaptation may facilitate the risk of secondary spillover, as already observed with mink in countries other than Denmark. 

This review of the zooanthroponotic aspects of SARS-CoV-2 reveals a number of important, yet unanswered, questions: 

To what extent is the burden of the human SARS-CoV-2 disease only a facet of the much larger epidemiological picture in nature? If we are unaware of an animal reservoir, where the virus circulates systematically, we may face unexpected secondary spillovers of novel or vanished strains, as observed in the emergence of mink cluster 5 in Denmark, or in the described cases of white-tailed deer and armadillo positivity for variants of concern that had ceased circulating in the community. 

Is there a similarly major viral effect in wild animal populations, one that we might understand retrospectively in the future? Are, for example, bat species threatened by potential spillback from humans in areas where SARS-CoV-2 (or its progenitor) was not previously present in bats [112]? North American bat populations, for example, are naive to sarbecoviruses, according to the results of epidemiological surveillance, and a potential spillback to them might have diverse consequences; the animals might be susceptible to the pathogen, exhibiting a disease that threatens their conservation, or the animals may serve as a viral reservoir capable of secondary spillover, or even recombination, for instance, with other coronaviruses that may lead to a novel pathogen with potential for human disease. 

What would be the effect of SARS-CoV-2 entrance in bonobo or mountain gorilla populations [113] and what would be the consequences for conservation? Will the experimental vaccines developed for animals be a solution to this conservation threat? We are still unaware of the overall efficacy of these animal vaccines. Moreover, we are unaware whether these vaccines will be equally efficient when dealing with novel SARS-CoV-2 variants. However, we do know that the sequential variants tending to prevail in the human population exhibit characteristics of increasing immune escape; this is a trajectory that was predicted all along [9] but is still evolving.

What is the proper model to describe the interplay between animal and human viral circulation for these kinds of pathogens? How does it affect the overall pandemic trajectory? We have already described how the spread of SARS-CoV-2 to mice might have resulted in the evolution of novel variants. One may also wonder how the pandemic would have progressed if a novel resistant variant, like mink cluster 5, had emerged unnoticed. 

Is it possible to envisage a “harmonious” co-existence of people with SARS-CoV-2 without addressing its parallel, undetected evolution through animals? 

More generally, how realistic is the goal of disease eradication when the transmission pathways involving spillbacks and second-generation spillovers are left out?

## Figures and Tables

**Table 1:** Animals (in alphabetical order) implicated in zooanthroponotic SARS-CoV-2 transmission and transmission importance.

Animals Involved	Extent ** of Transmission	Secondary Spillover *	Scientific and/or Common Names *** of the Species/Subspecies Reported—Comments
Anteater	+		[*Myrmecophaga tridactyla*, Giant anteater] Single case of an injured individual found RT-PCR positive in Brazil [12].
Armadillo	++		[Chaetophractus villosus, Big hairy armadillo] Numerous positive individuals in Argentina, moreover, with variants of concern that have long ceased to circulate in humans [13].
Badger	+		[*Meles meles*] Antibodies positive in two out of ten individuals in France, randomly sampled [14].
Beaver	+		[*Castor fiber*, Eurasian beaver] Seven individuals reported as infected from workers in a beaver-breeding facility in Mongolia [15].
Binturong	+		[*Arctictis binturong*] An asymptomatic case and a symptomatic case in the Illinois zoo outbreak [16].
Camels	+		High antibody positivity (71%) in a study from Kenya [17].
Cats	+++	+	[Domestic cat] Epidemiology extensively studied in household, stray, and shelter animals; recently reviewed [18]; see also the relevant chapter “what our pets taught us”.
Cattle	+		A total of 11 out of 1000 seropositive animals in Germany, 14 out of 24 in Italy, considered as random events [19,20].
Coati	+		[*Nasua nasua*, White-nosed coati] Two asymptomatic cases in the Illinois zoo outbreak [16]. [South American coati] Two out of forty-four randomly sampled RNA-positive in Brazil [21].
Deer	++++	+?	[*Odocoileus virginianus*, White-tailed deer] SARS-CoV-2 is prevalent in this deer species in North America; see relevant chapter “what the deer taught us”. [*Odocoileus hemionus*, Mule deer] One isolated case in Utah [22].
Dog	++	+?	See relevant chapter “what our pets taught us”.
Ferret	++		[*Mustela putorius furo*, Pet/Domestic ferret] Multiple animals positive in Spain [23]; case report of human to animal transmission also from Slovenia [24] and the US [21].
Fishing cat	+		[*Prionailurus viverrinus*] A symptomatic case in the Illinois zoo outbreak [16].
Fox	+		[*Vulpes vulpes*} A case in red foxduring surveillance testing in Switzerland [25]
Gorilla	++		[*Gorilla gorilla gorilla*, Western lowland gorilla] Captive animal clusters reported from US and the Czech Republic. Initially, at a San Diego zoo [26], then, at least four cases in a Georgia zoo (although 18 out of 20 animals were symptomatic) [27], and five cases in a population of eight, infected from a zoo keeper and subsequently transmitted from animal to animal, in the Prague Zoological Garden [28]. Another cluster of cases has recently been reported from a Spanish zoo [29].
Hamster	+++	+	[*Mesocricetus auratus*, Golden Syrian hamsters] See relevant chapter “what the rodents taught us”, on spillback and international secondary spillover event. Experimentally, extremely prone to infection [30].
Hippopotamus	+		Two cases with mild symptoms in the Royal Zoo of Antwerp, Belgium [31].
Hyena	+		[*Crocuta crocuta*, Spotted hyena] Two cases in Colorado zoo [32].
Leopard	+		[*Panthera bengalensis euptilurus,* Amur leopard cat] in the Prague zoo outbreak [28]. [*Panthera uncia*, Snow leopard] Early (2020) zoo cluster of three individuals in Kentucky [33]; individuals infected in the Illinois zoo outbreak [16]. [*Panthera pardus fusca*, Indian leopard] Single fatal case reported [34].
Lion	+	+?	[*Panthera leo*, African lion] Numerous zoo clusters, including the first zoo SARS-CoV-2 incident, in Bronx [35]; other clusters in a Johannesburg zoo [36] and a Barcelona zoo [*Panthera leo bleyenberghi*, Southwest African Lion] [37]. [*Panthera leo persica*, Asiatic lion] Clusters in at least three Indian zoo facilities [38,39]. [Unspecified lion] in a Singapore zoo [32].
Lynx	+		[*Lynx canadensis*, Canadian lynx] A single zoo case in Pittsburgh. [*Felix lynx*, Eurasian lynx] A single zoo case in Zagreb, Croatia [40].
Manatee	+		[*Trichechus manatus manatus*, Antillean manatee] Animals were sampled in a conservation facility in Brazil; two out of nineteen individuals positive [41].
Mandrill	+		A single case in a US zoo, mild symptoms [41].
Marmoset	+		[*Mico melanurus*, Black-tailed marmoset] A single Brazilian case report for a free-ranging animal, the first in a New World monkey [42].
Marten	+		[*Martes martes*, European pine marten] Antibodies positive in three out of fourteen individuals in France, randomly sampled [14].
Mink	++++	+	[*Neovison vison*, American mink] See relevant chapter “what the mink taught us”.
Monkey	+		[Common squirrel monkey] A single case in a US zoo [43].
Otter	+		[*Aonyx cinereus*, Asian small-clawed otter] Outbreak reported in an aquarium in Georgia, US [44]. [*Lutra lutra*, Eurasian river otter] Single case report in Spain [45].
Puma	+		Two reports in a South African zoo, in different time periods [36].
Rabbits	+?		Limited antibody positivity in a study of 144 pet rabbits (two positives) [46].
Tiger	++	+?	[*Panthera tigris*] Numerous captive tiger zoo clusters, in Bronx zoo [35]. [*Panthera tigris jacksoni*, Malayan tiger] One cluster in Bronx zoo [34]; reports also in Tennessee and Virginia [47,48]. [*Panthera tigris altaica*, Amur tiger] One cluster in Bronx zoo [35]. [*Panthera tigris sumatrae*, Sumatran tiger] Two cases in a Jakarta zoo, Indonesia [49], one case in the Prague zoo outbreak [28].

* See text for details of secondary animal to human transmission for each animal implicated. ** Extent of transmission rating: + isolated case reports, ++ several case reports or small clusters, +++ several transmission reports with potential for secondary generation spillover or extended intraspecies transmission, ++++ documented extended intraspecies transmission and/or secondary generation spillover. *** Names of animals are as given in the associated literature sources.
